# SLUG: a new target of lymphoid enhancer factor-1 in human osteoblasts

**DOI:** 10.1186/1471-2199-11-13

**Published:** 2010-02-03

**Authors:** Elisabetta Lambertini, Tiziana Franceschetti, Elena Torreggiani, Letizia Penolazzi, Antonio Pastore, Stefano Pelucchi, Roberto Gambari, Roberta Piva

**Affiliations:** 1 Department of Biochemistry and Molecular Biology, Molecular Biology Section, University of Ferrara, Via Fossato di Mortara, 74, 44100 Ferrara, Ferrara, Italy; 2 ORL Division, University of Ferrara, Ferrara, Italy; 3 Department of Reconstructive Science, University of Connecticut Health Center, Farmington, Connecticut, USA

## Abstract

**Background:**

Lymphoid Enhancer Factor-1 (Lef-1) is a member of a transcription factor family that acts as downstream mediator of the Wnt/β-catenin signalling pathway which plays a critical role in osteoblast proliferation and differentiation. In a search for Lef-1 responsive genes in human osteoblasts, we focused on the transcriptional regulation of the SLUG, a zinc finger transcription factor belonging to the Snail family of developmental proteins. Although the role of SLUG in epithelial-mesenchymal transition and cell motility during embryogenesis is well documented, the functions of this factor in most normal adult human tissues are largely unknown. In this study we investigated SLUG expression in normal human osteoblasts and their mesenchymal precursors, and its possible correlation with Lef-1 and Wnt/β-catenin signalling.

**Results:**

The experiments were performed on normal human primary osteoblasts obtained from bone fragments, cultured in osteogenic conditions in presence of Lef-1 expression vector or GSK-3β inhibitor, SB216763. We demonstrated that the transcription factor SLUG is present in osteoblasts as well as in their mesenchymal precursors obtained from Wharton's Jelly of human umbilical cord and induced to osteoblastic differentiation. We found that SLUG is positively correlated with RUNX2 expression and deposition of mineralized matrix, and is regulated by Lef-1 and β-catenin. Consistently, Chromatin Immunoprecipitation (ChIP) assay, used to detect the direct Lef/Tcf factors that are responsible for the promoter activity of SLUG gene, demonstrated that Lef-1, TCF-1 and TCF4 are recruited to the SLUG gene promoter "*in vivo*".

**Conclusion:**

These studies provide, for the first time, the evidence that SLUG expression is correlated with osteogenic commitment, and is positively regulated by Lef-1 signal in normal human osteoblasts. These findings will help to further understand the regulation of the human SLUG gene and reveal the biological functions of SLUG in the context of bone tissue.

## Background

Lymphoid Enhancer binding Factor-1 (Lef-1) is a nuclear high mobility group (HMG) protein that mediates gene transcription in response to canonical Wnt/β-catenin signaling pathway [1-3]. Wnt signaling controls normal and abnormal development in a variety of tissues including skeleton, and accumulated evidence has shown that Lef-1 influences osteoblast proliferation, maturation, function, and regeneration both *in vitro *and *in vivo *[4-7]. Nevertheless, the exact mechanism by which Lef-1 affects osteoblast differentiation is unknown. In a search for Lef-1 responsive genes in human osteoblasts, we focused on the transcriptional regulation of the SLUG gene for the reasons reported below.

SLUG, also named SNAIL2, is a member of a superfamily of zinc-finger transcription factors that play a central role in the patterning of vertebrate embryos [8-10]. It is implicated in the induction of epithelial mesenchymal transitions (EMT) at specific stages of normal development and tumor progression, acting as a transcriptional repressor of genes encoding components of cell-cell adhesive complexes in the epithelia [11-17]. Several signalling pathways inducing EMT cellular event and including FGF, WNT, TGF-β, BMP, EGF, HIF, Notch, PTH, integrins and SCF/c-Kit have been shown to converge in SNAIL genes induction, as well reviewed by Barrallo-Gimeno et al. [18], and as previously reported [9,10,19].

SLUG and its family members also have important roles in other processes, including protection of cells from programmed cell death, regulation of cytoskeletal elements [18], adipocyte differentiation [20] and migration of neural crest cells [21,22]. Although the expression of SLUG has been found in most normal adult human tissues [23-25], little is known about its potential functions.

It is important to underline that the vertebrate neural crest, formed at the border between the neural plate and the non-neural ectoderm during neurulation, is able, under SLUG control, to give rise to different cell types including neurons, glia, facial chondrocytes, osteoblasts, and melanocytes [8,26,27]. In addition, craniofacial abnormalities have been observed in association with cerebral malformations and cutaneous lesions in some neurocutaneous syndromes, emphasizing an important inductive role of the neural tube in the development of non-neural tissues mediated through neural crest and differentiating genes such as SLUG and Sox10 [28,29]. Overall, these observations encourage investigation on SLUG expression and functions in adult cells, including osteoblasts.

We recently demonstrated, by a knockdown approach, that SLUG is involved in the differentiation and maturation process of normal human osteoblasts [30]. Nevertheless, so far, no data have been presented on SLUG regulation in these cells and their precursors. Only one previous investigation has demonstrated that Wnt signaling regulates SLUG expression, in a tumor model, such as an osteosarcoma cell line, mediating cancer invasion [31].

The presence of putative *cis *elements for Lef-1, in human SLUG gene promoter has raised the possibility that Lef-1 may be implicated in the modulation of SLUG expression as previously demonstrated in other species such as chick and Xenopus [32,33]. In this study we demonstrated that SLUG is expressed in both normal human osteoblasts and their mesenchymal precursors, and that Lef-1 is recruited "*in vivo*" to its promoter acting as a positive transcriptional regulator.

## Results

### SLUG expression in human osteoblasts and their mesenchymal precursors

**Figure 1:**
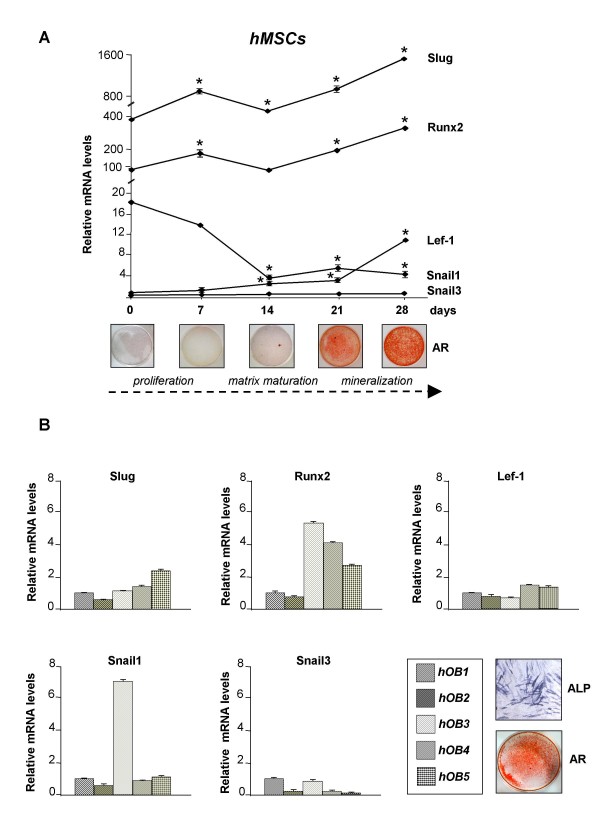
**Detection of SLUG expression by quantitative RT-PCR**. The level of SLUG, RUNX2 Lef-1, SNAIL1 and SNAIL3 expression was examined by quantitative RT-PCR in three hMSC samples cultured up to 28 days in osteogenic medium (A) and in five hOB samples (B). The cDNA obtained from total RNA was subjected to quantitative TaqMan RT-PCR for SLUG, RUNX2, Lef-1, SNAIL1 and SNAIL3 transcript analysis. The experiments were carried out in triplicate, the expression levels were normalized on the basis of GAPDH expression and results of the experiments are reported as relative mRNA expression levels. ΔΔCt method was used to value the gene expression; standard error of the mean (SEM) was calculated. The commitment to osteoblastic lineage of hMSCs was evaluated by Alizarin Red staining for extracellular calcium deposition. The authentic osteoblast phenotype was confirmed in hOBs by staining for alkaline phosphatase (ALP) activity and mineralized matrix deposition (AR, Alizarin Red staining). * = p < 0.05 (respect to day 0).

Lef-1 has been shown to play a role in osteoblast differentiation and function. Owing to the relationship between Lef-1, β-catenin and SLUG recently found in some epithelial-mesenchymal transition cellular models [34,35], we hypothesized that Lef-1 and SLUG may also be correlated in osteoblast lineage cells. To test this idea SLUG expression was examined during osteoblast differentiation and compared with Lef-1 expression levels. SLUG mRNA levels were measured in human mesenchymal stem cells (hMSCs) obtained from umbilical cord Wharton's Jelly and induced towards osteogenesis, as previously described [36]. RNA was collected after 0, 7, 14, 21, and 28 days in culture and evaluated by quantitative RT-PCR. As shown in Figure 1A, these cells differentiate along the osteoblast lineage in osteogenic medium as confirmed by the positive staining for extracellular calcium deposition. Abundant SLUG mRNA was detected in the cells at all times tested, and was induced as the cultures progressed. Lef-1 was less abundant, but significantly increased during the osteogenesis. RUNX2, a determinant transcription factor for osteoblastogenesis [37], was also expressed at all stages, and was induced as the cultures progressed, confirming that each time point represented increasingly mature osteoprogenitors.

In order to confirm that the expression profile that we found was associated with osteoblast phenotype, SLUG, Lef-1 and RUNX2 expression levels were measured in human primary osteoblasts obtained from five bone specimens (hOBs). All these samples were positive for alkaline phosphatase (ALP) activity, a well-known osteoblast differentiation marker, and were able to form mineralized nodular structures after 14 days in osteogenic condition (see a representative experiment in the panel of Figure 1B). As shown in Figure 1B, SLUG, Lef-1 and RUNX2 were detected in all hOB samples analyzed. The level of SLUG mRNA in hOBs was also compared with that found in different osteoblast-like cell lines [Additional file 1].

To further characterize the potential involvement of SNAIL family members in osteogenesis, the expression of SNAIL1 and SNAIL3 was examined in the same set of experiments. SNAIL1 has been recently reported to act on the osteoblast population regulating bone cells differentiation and contributing to bone remodeling in mice [38]. In agreement with this previous study, we found that SNAIL1 was expressed at early stages of osteoblast differentiation and then downregulated for differentiation to proceed (Figure 1A). In hOB samples SNAIL1 was expressed at substantial levels (Figure 1B). The expression of SNAIL3 [39] was detectable at very low levels in the hMSCs induced towards osteogenesis (Figure 1A), and at low levels in hOBs (Figure 1B).

### SLUG expression is positively modulated by Lef-1

**Figure 2:**
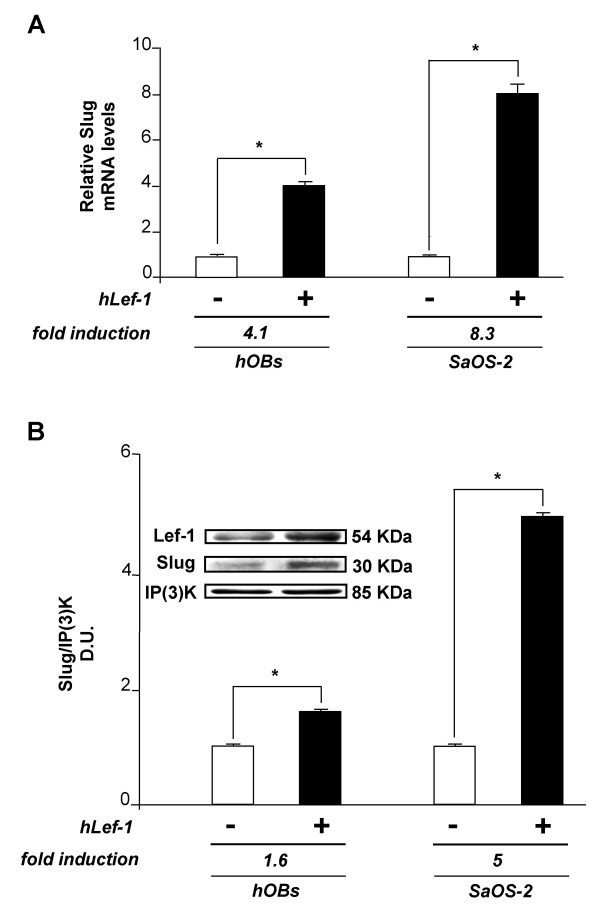
**Effect of Lef-1 overexpression on SLUG expression in hOBs**. The effect of Lef-1 overexpression was examined at mRNA (A) and protein (B) level. (A) SLUG mRNA was evaluated by quantitative RT-PCR in hOBs and SaOS-2 osteoblast-like cells transfected with 2.5 μg of hLef-1 (K14-myc-hLEF1) expression plasmid. The cDNA obtained from total RNA was subjected to quantitative TaqMan RT-PCR for SLUG transcript analysis. The expression levels were normalized on the basis of GAPDH expression and results of the experiments are reported as relative mRNA expression levels. Results are representative of three independent experiments carried out in triplicate. ΔΔCt method was used to compare gene expression data; standard error of the mean (SEM) was calculated. * = p < 0.05. (B) SLUG protein levels were examined by Western blot analysis in hOBs and SaOS-2 osteoblast-like cells transfected with 2.5 μg of hLef-1 expression plasmid. Whole cell lysates were prepared and 25 μg of protein run on a 12% SDS-polyacrylamide gel. The proteins were visualized using Supersignal West Femto Substrate (Pierce). The quantitative presentation of the protein levels were performed by densitometric analysis using Anti-IP(3)K as control. D.U. = densitometric units. This experiment was repeated three times with similar results. A representative SLUG and Lef-1 Western blot analysis with size markers (KDa) is reported. * = p < 0.05.

hOBs were then transfected with expression vector containing hLef-1 cDNA (K14-myc-hLEF1) as described in the Methods section. As shown in Figure 2, SLUG expression significantly increased in Lef-1 overexpressing cells, both at mRNA and protein level, as demonstrated by RT-PCR (Figure 2A) and Western blot analysis (Figure 2B). The significant increase of Lef-1 in the cells transfected with hLef-1 expression vector was confirmed by the same Western blot analysis (Figure 2B). As expected, forced expression of Lef-1 increased Slug expression to higher levels in SaOS-2 osteoblast-like cells than in hOBs, because of a higher intrinsic transfection facility of this cell line.

**Figure 3:**
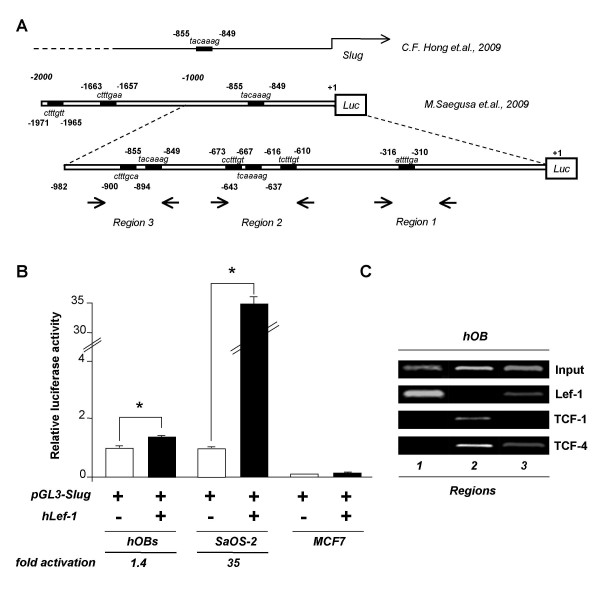
**Lef-1 affects the activity of human SLUG promoter "*in vitro*" and binds it "*in vivo*"**. (A) The SLUG promoter region under investigation is reported. The positions of putative Lef/Tcf binding sites are enclosed by rectangles and are compared with those recently investigated by others. Positions of PCR primers used in ChIP experiments are also reported. (B) The DNA construct containing the human SLUG promoter region was cloned into upstream of the firefly luciferase (LUC) reporter gene. hOBs and SaOS-2 osteoblast-like cells were transfected with the pGL3-SLUG Luc reporter vector containing the sequence from +1 to -982 of the human SLUG promoter (pGL3-SLUG 982 bp), in the absence (-) or presence (+) of 2.5 μg of hLef-1 expression plasmid. The results of reporter gene assays were normalized with protein concentration and β-gal activity for transfection efficiency and the data are represented as ratios of luciferase units to β-galactosidase units. MCF7 breast cancer cell line was used as negative control. All experiments were performed in triplicate and the average of the ratio of the reporter activity + SEM is shown. * = p < 0.05. (C) Recruitment of Lef1/TCF transcription factors to the human SLUG promoter is demonstrated by "*in vivo*" chromatin immunoprecipitation (ChIP) binding assays. Soluble chromatin was prepared from hOBs and immunoprecipitated with the indicated specific antibodies against Lef-1, TCF-1, and TCF-4. The associations of the transcription factors to bound precipitated DNA were monitored on the human SLUG promoter regions 1, 2 and 3 by PCR with the primers indicated in the scheme. Input represents a positive control using the starting material (0.2%) prior to immunoprecipitation. Representative agarose gels are shown.

The ability of Lef-1 to activate transcription of SLUG gene was then tested on the human SLUG promoter (Figure 3). We chose to focus on an approximately 1 Kb fragment upstream of the transcription start site in the SLUG gene since it contains sequences involved in the regulation of promoter activity mediated by β-catenin [34]. In addition to the previously identified TCF binding site at -859/-855 position [34,35], we identified, in this region, another five potential consensus binding sites for the Lef/Tcf family by using the programs Transcription Element Search Software TESS for transcription factor search and MatInspector 7.4 program (Figure 3A). The sequence was cloned upstream of the Luc reporter gene in the pGL3basic vector, and the construct, (named 982 bp luc-construct), was assayed after osteoblast transfections performed with or without Lef-1 expression plasmid. As shown in Figure 3B, transient transfection with the luciferase reporter 982 bp luc-construct resulted in an increase in luciferase activity relative to the empty, promoterless pGL3-basic vector, demonstrating that this DNA fragment contains significant promoter activity in hOBs (5-10 fold increase). Co-transfection with plasmid encoding Lef-1 produced a significant increase in Luc activity as compared with cells containing the 982 bp luc-construct reporter plasmid. This increase was dramatic in Lef-1 overexpressing SaOS-2 cells. On the contrary, the same experiments performed in the non-osseous SLUG-negative MCF7 breast cancer cell line revealed no promoter activity.

As a whole, these data indicate that Lef-1 upregulates SLUG gene expression in normal human osteoblasts.

### Lef-1 is recruited to the SLUG promoter "in vivo"

Next, we investigated whether Lef-1 could, "*in vivo*", physically bind with the human SLUG promoter. Considering that in addition to Lef-1, among TCF family members, both TCF-1 and TCF-4 are expressed in osteoblasts [2], the analysis was addressed to all three proteins. The binding of transcription factor to the SLUG promoter was verified by performing *in vivo *chromatin immunoprecipitation (ChIP) assays (Figure 3C). To this aim, hOBs were exposed to formaldehyde to cross-link proteins and DNA, and were sonicated to fragment the chromatin. Specific antibody against Lef-1, TCF-1 and TCF-4 were used to immunoprecipitate the protein-DNA complexes. After immunoprecipitation, DNA was extracted from the beads and used as a template to generate specific PCR products. The presence of the promoter specific DNA region before immunoprecipitation was confirmed by PCR (input). In the SLUG promoter fragment used for the reporter assay, three different regions were identified, as depicted in Figure 3A, and analyzed by a set of primers spanning the six consensus binding sites for the Lef/Tcf family. The amplified product sizes (bp) were 178 for region 1, 164 for region 2, and 165 for region 3. The results showed that the promoter region 3, containing the previously identified TCF binding site at -859/-855 position [34,35], was significantly immunoprecipitated by Lef-1 and TCF4 antibodies, but that Lef-1 was mostly associated with the promoter region 1 and not at all with the promoter region 2 (Figure 3C). On the contrary, we found that region 2 was rather occupied by TCF-1 and TCF-4. Therefore, the observation that the endogenous SLUG gene expression may be increased by Lef-1 was further validated by the *in vivo *occupancy of the Lef/TCF regulatory sites in the SLUG gene promoter.

### Activation of Wnt signaling by GSK-3β inhibitor increases SLUG promoter activity

**Figure 4:**
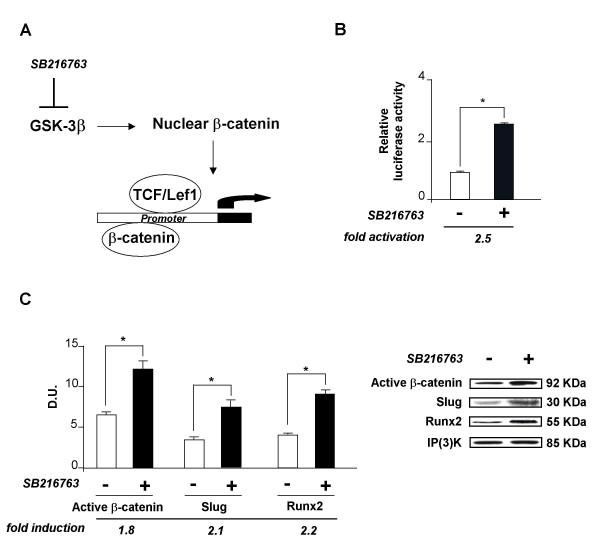
**Treatment of hOBs with the glycogen synthase kinase (GSK-3β) inhibitor, SB216763**. (A) A scheme of SB216763 action mechanism is reported (see the text for details). (B) Effect of SB216763 on the TOPflash reporter system. 24 h after transient transfection with the TOPflash plasmid, the cells were treated (+) or not (-) with SB216763 (10 μM) for 24 h prior to harvest. Luciferase activity was normalized to β-galactosidase activity in the same sample. The bars represent mean ± SEM. * = p < 0.05. (C) The levels of β-catenin expression, SLUG and RUNX2 were examined by Western blot in hOBs treated with SB216763 (10 μM) or with the only vehicle (-). The quantitative presentation of the protein levels was performed by densitometric analysis using Anti-IP(3)K as control. D.U. = densitometric units. A representative Western blot analysis with size markers (KDa) is reported. * = p < 0.05.

It has been demonstrated that β-catenin promotes Lef/Tcf interaction with target DNA sequence in many cellular contexts. In order to support the role of Lef/Tcf transcription factors in SLUG expression regulation, we next investigated whether β-catenin activation was involved in SLUG expression regulation. We used a treatment with SB216763 as a model for β-catenin activation (Figure 4A). This compound binds and specifically inhibits glycogen synthase kinase GSK-3β. GSK-3β is a serine/threonine kinase, originally identified as a kinase that is involved in glucose metabolism, but recent research has determined that it acts on a wide variety of substrates, including transcription factors, and is a key regulator in many signalling pathways [40]. This enzyme is known to be a key negative regulator of canonical Wnt/β-catenin and PI3K/Akt signalings [41]; hence, its inhibition activates Wnt signalling selectively via the β-catenin/TCF pathway and results in relocation of stabilized β-catenin to the nucleus. As expected, the SB216763-treated cells transfected with the β-catenin/Tcf transcription reporter construct -TOPflash reporter system- showed an increase in TOPflash activity up to 4-fold (Figure 4B). The β-catenin/Tcf transcription reporter assay was recognised as an important assessment method for evaluation of the Wnt pathway activity. As TOPflash has three TCF-binding sites, it could be applied to represent the activation of the Wnt pathway. In fact, our data showed that SB216763 treatment positively affected β-catenin expression, as revealed by Western blot reported in Figure 4C. The dose- and time-response to SB216763 cell treatment was analyzed in terms of SLUG mRNA levels in osteoblast-like cell lines [Additional file 2]. The same analysis demonstrated that the increase in β-catenin mediated by SB216763 was correlated with a significative increase in SLUG and RUNX2 expression both at protein (Figure 4C) and mRNA level (data not shown). Therefore, on the whole, this suggests that the canonical Wnt signaling positively affects SLUG expression in normal human osteoblasts via the β-catenin/TCF pathway because, by potentiating β-catenin, SLUG expression increases.

## Discussion

In this paper we have demonstrated that the transcription factor SLUG is present in normal human osteoblasts and their mesenchymal precursors. Osteoblasts are the primary cell type responsible for the bone remodeling process, and alterations in this pathway can lead to osteopenic disorders such as osteoporosis. Therefore, any new marker or mechanism associated with differentiation of these cells represent very relevant information for the study of bone biology and bone-related diseases in general.

We have shown that SLUG expression increases during osteogenesis, and is positively regulated by Lef-1, an osteoblastic transcription factor which we found *in vivo *recruited by specific *cis *elements present in the SLUG promoter. In the SLUG promoter region of approximately 1 Kb upstream of the transcription start site, we found at least six potential consensus binding sites for the Lef/Tcf family, and not just one only at -859/-855 position, as recently reported [34,35]. We found that the sequence regions containing these sites are all involved, even if at different levels, in the *in vivo *recruitment of Lef/Tcf factors, including Lef-1, TCF-1 and TCF-4, in human osteoblasts. The investigations on the only previously characterized TCF binding site (-859/-855), demonstrated its ability to recruit TCF-4 in SW480 human colon cancer [35], but not in Hec251 endometrial cancer cell line [34] where, on the contrary, SLUG expression seems to be under transcriptional control of β-catenin without the binding of Lef/Tcf factors at this site. Other studies in different experimental models provide evidence that Xenopus and mouse SLUG promoters are directly activated by β-catenin/TCF complexes through the binding sequences [32,33], and that SLUG promoter activity may be inhibited by dominant negative Tcf [42]. Combined with these reports, our results may lead to the hypothesis that, directly or indirectly, SLUG and Lef-1 are strictly correlated in many cellular events, including osteoblast differentiation, mediated by Wnt/β-catenin signalling. In addition, this is supported by our recent evidence demonstrating the requirement of SLUG for osteoblast maturation and the decrease in Wnt/β-catenin signalling after SLUG knockdown [30]. This suggests a possible role of SLUG as effector of Wnt/β-catenin signalling.

Our findings confirm a relationship between SLUG and Wnt signalling showing that the increase in β-catenin levels, obtained by the suppression of GSK-3β activity with SB216763 inhibitor, induces a significative SLUG gene expression increase. β-catenin is known to associate with the Lef/Tcf transcription factor family and promote the expression of several genes through the recruitment of other factors to form a transcriptionally active complex [43,44]. Lef-1 is reported to have an important role in osteoblast maturation for its ability in the regulation of expression of genes involved in the stimulation of bone formation, such as RUNX2 and Col11a1 [45,46]. In addition, an age- and gender- dependent role for Lef-1 in regulating bone formation *in vivo *has recently been described [7]. The discovery that SLUG expression is upregulated during osteogenesis, is positively correlated with the expression of RUNX2 and Lef-1, and is under the control of Lef-1, corroborates the role of Lef/Tcf transcription factors in osteoblasts and highlights mechanisms by which Lef-1 may affect maturation and differentiation of these cells. Our results further support the hypothesis that SLUG may have a distinct role in normal human osteoblasts, and may be positively regulated by activity of canonical Wnt/β-catenin signalling pathway. Therefore, as far as bone tissue is concerned, SLUG should not be considered exclusively as a marker of malignancy and an attractive target for therapeutic modulation of bone metastasis and osteosarcoma invasiveness, as indirectly suggested by Guo et al. [31].

Considering the widespread expression of SLUG in all osteoblast samples analyzed, we cannot exclude that SLUG, which encodes an evolutionarily conserved antiapoptotic transcription factor, may confer a survival advantage in osteoblasts, as demonstrated for leukemic B cell progenitors [47].

In conclusion, although further studies are required to elucidate whether the two Lef-1 isoforms recently identified [6] may have distinguishable activities in determining the proper levels of SLUG expression, our study clearly shows that β-catenin/Lef-1 signalling is involved in the regulation of this gene in normal human osteoblasts. In addition, other factors may contribute to the SLUG gene regulation. At present, the relationship between other *cis*-regulatory elements in the SLUG promoter and osteoblast-inducing signals is completely unknown. The most likely candidates for this function are SLUG and RUNX2, which could associate with the E boxes and RUNX binding sites present in the promoter. Therefore, further work will be necessary to evaluate a potential transcription autoregulation and to elucidate the association between SLUG and RUNX2 expression. Our hypothesis is that SLUG might represent an interesting molecule for normal skeletogenesis acting inside the recently proposed [48] large signalosome in which inputs from Wnt/β-catenin/Lef-1 signalling, steroid receptors, BMPs, and kinases converge to induce differentiation of osteoblast precursors. With this in mind, we also speculate that study of the association between SLUG and some organizers of osteoblastic phenotype may improve the characterization of the human osteoblast differentiation stages. In particular, this may be relevant in approaches addressed to the discovery of new molecular targets to use in bone repair and regenerative medicine.

## Conclusions

In this study we showed that transcription factor SLUG is expressed in both normal human osteoblasts and their mesenchymal precursors, and that Lef-1, a mediator of the Wnt/β-catenin signalling pathway, is recruited "*in vivo*" to its promoter acting as a positive transcriptional regulator. The relationship between SLUG and Wnt signalling has been confirmed demonstrating that increase in β-catenin levels induced a significative SLUG gene expression increase.

In conclusion, our findings reveal the biological functions of SLUG in the context of bone tissue showing that it is positively correlated with the osteogenesis, and highlights mechanisms by which Lef-1 may affect maturation and differentiation of osteoblasts.

## Methods

### Construction of reporter plasmid

Promoter region (+1 to -982 bp) of the human SLUG promoter was amplified by PCR from human genomic DNA using SLUG F genomic primer as sense primer and SLUG R genomic primer as antisense primer (Table 1). The PCR product was subcloned upstream of a firefly luciferase (LUC) gene in the promoter-less pGL3-Basic vector (Promega, Madison, WI) using MluI and BglII restriction sites, and the presence of the insert was confirmed by restriction digestion.

**Table 1:** Primers used in this study

*Oligo name*	*Primer sequences 5'-3'*
***Primers for reporter construct***	
Slug F	TGTCAAAAGTGTGAGAGAAT
Slug R	CTTGCCAGCGGGTCTGGC

***Primers for ChIP***	
Region 1 F	GAGGTTACCTCTCTTGAAAATACT
Region 1 R	GGAAGAAAGATCCAATCACA
	
Region 2 F	CCAGGCCAGATCCCAGGAGAGC
Region 2 R	GCCTCTGGTGTTAATGAGAGCCTA
	
Region 3 F	TGCCCCCCTTCTCTGCCAGAGTT
Region 3 R	TTCCGCGAAGCCAGGGGCAGCG

The sequence and the name of the forward (F) and reverse (R) primers for the construction of reporter plasmid and for Chromatin immunoprecipitation (ChIP) are reported.

### Cell culture, plasmids and transient transfection

Human primary osteoblasts were obtained from bone samples collected during nasal septum surgery, and were cultured as previously described [49]. Recruitment of subjects donating osteoblasts was in accordance with approved procedures, and informed consent was obtained from each patient. Briefly, the bone was cut into small pieces which were rinsed and then cultured in Eagle's MEM (Sigma Aldrich, St. Louis, MO, USA) supplemented with 20% fetal bovine serum (FBS) (CELBIO EuroClone, Milan, Italy), 2 mM glutamine, 100 units/ml penicillin, 100 μg/ml streptomycin, and 50 μg/ml ascorbate at 37°C in a humidified atmosphere of 5% CO_2_. After about 5-7 days, outgrowth of bone cells from the bone chips commenced, and confluency in 9 cm^2 ^dishes was usually reached after 4-6 weeks. For the studies here presented, only first passage cells were used.

Mesenchymal stem cells were obtained from Wharton's Jelly of human umbilical cord after the mothers' consent and approval of the "Ethical committee of University of Ferrara and S.Anna Hospital ", and characterized as previously described [36].

The expression vector for full-length Lef-1 (K14-myc-hLEF1) was a gift from Elaine Fuchs and Rebecca C. Lancefield (Howard Hughes Medical Institute, The Rockfeller University, Lab. of Mammalian Cell Biology & Development, New York U.S.A.). The TCF reporter plasmid TOP FLASH was kindly provided by Rolf Kemler (Max Planck Institute, Heidelberg, Germany).

For transient transfection assays, 50000 cells/ml were seeded in 24 or 6 multiwell plates. After 24 h, cells were transfected using Lipofectamine reagent (Invitrogen, Carlsbad, CA) and 0.5 μg of reporter construct where not specified.

SB216763 was purchased from Sigma (Sigma Aldrich, St. Louis, MO, USA), and dissolved in DMSO.

### Analysis of the osteoblast phenotype

For alkaline phophatase staining, prefixed mono-layered cells were incubated at room temperature in a solution containing naphthol AS-BI phosphate and freshly prepared fast blue BB salt buffered at pH 9.5 with 2-amino-2-methyl-1,3-propanediol (Alkaline Phosphatase Leukocyte kit, Sigma). The presence of sites of ALP activity appeared as blue cytoplasmatic staining.

The extent of mineralized matrix in the plates was determined by Alizarin Red S staining (Sigma) in the cells cultured for up to 35 days in osteogenic medium consisting in DMEM, high-glucose, supplemented with 10% FBS, 10 mM β-glycerophosphate, 0.1 mM dexamethasone and 50 mM ascorbate. In the committed cells, the osteogenic medium was changed every three days. The cells were then fixed in 70% ethanol for 1 h at room temperature, washed with PBS, stained with 40 mM AR-S (pH 4.2) for 10 min. at room temperature, washed five times with deionized water and incubated in PBS for 15 min. to eliminate non-specific staining. The stained matrix was observed at different magnifications using a Leitz microscope.

### Luciferase reporter gene assays

For experiments assessing activation of the SLUG promoter, 1 μg of reporter plasmid was cotransfected with 2.5 μg of expression vectors for Lef1 (K14-myc-hLEF1) and 0.25 μg of pCMV-Sport-βgal (Invitrogen). The cells were lysed 48 h after transfection using the reporter lysis buffer (Promega, Madison, WI). Luciferase and β-galactosidase activities were determined with luciferase and Beta-Glo assay systems respectively (Promega, Madison, WI). Their activities were normalized with respect to total protein amount.

### Real-time RT-PCR analysis

For mRNA analysis total cellular RNA was extracted using Total RNA Isolation System (Promega) and cDNA synthesis was performed for 1 h at 42°C using 1 μg of total RNA as a template and 100 U of reverse transcriptase ImProm-II (Promega) as previously described [49]. The level of mRNA expression was analyzed by quantitative real-time PCR using the ABI Prism 7700 system (Applied Biosystems) and the following TaqMan MGB probes: 5' FAM-ATGATGAAAGGTGGGATACGAAAAG-TAMRA 3' for SLUG, 5' FAM-GAACCCAGAAGGCACAGACAGAAG-TAMRA 3' for RUNX2, 5' FAM-TCTAATCCAGAGTTTACCTTCCAGC-TAMRA 3' for SNAIL1, 5'FAM-GAGACGCAGAGAGAAATCAATGGTG-TAMRA 3' for SNAIL3, and 5' FAM-CATGTCCAGGTTTTCCCATCATATG-TAMRA 3' for Lef-1; GAPDH mRNA was used as an endogenous control (Applied Biosystems Inc, Foster City, CA, USA) and quantification was performed using a TaqMan assay. The mRNA levels of target genes were corrected for GAPDH mRNA levels. All PCR reactions were performed in triplicate for each sample and were repeated three times. All experimental data were expressed as the mean ± SEM.

### Western Blot analysis

For Western Blot analysis, the cells were washed twice with ice-cold PBS and cell lysates were prepared as previously reported [50]. 25 μg of each sample was then electrophoresed on a 12% SDS-polyacrylamide gel. The proteins were then transferred onto an Immobilon-P PVDF membrane (Millipore Corporation, 900 Middlesex Tpk Billerica, USA). After blocking with PBS-0.05% Tween 20 and 5% dried milk, the membrane was probed with the following antibodies: SLUG (L40C6) from Cells Signaling Technology Inc. (Danvers, CA, USA), RUNX2 (sc-10758) from Santa Cruz Biotechnology (Santa Cruz, CA), Lef-1 (L7901) from Sigma Aldrich (St Luis, MO, USA), IP3K (06-195) and Active-β-catenin from Upstate Biotechnology, Inc. (Lake Placid, NY). After washing with PBS-Tween, the membranes were incubated with peroxidase-conjugated anti-rabbit antibody (1:50000) or anti-mouse (1:2000) (Dako, 2600 Glostrup, Denmark) in 5% non-fat milk. Immunocomplexes were detected using Supersignal West Femto Substrate (Pierce). Anti-IP(3)K was used to confirm equal protein loading.

### Chromatin immunoprecipitation (ChIP) assay

The ChIP assay was carried out as previously described [49] using the standard protocol supplied by Upstate Biotechnology (Lake Placid, NY) with their ChIP assay reagents.

The cells were cross-linked with 1% formaldehyde for 10 min at 37°C, washed in ice-cold PBS and resuspended in SDS lysis buffer for 10' on ice. Samples were sonicated, diluited 10-fold in diluition buffer supplemented with protease inhibitors and precleared with 80 μl of DNA-coated protein A-agarose; the supernatant was used directly for immunoprecipitation with 5 μg of anti- Lef-1 (sc-8591), TCF-1 (sc-13025) and TCF-4 (sc-13027) (Santa Cruz Biotec, Ca, USA) overnight at 4°C. Immunocomplexes were mixed with 80 μl of DNA-coated protein A-agarose followed by incubation for 1 h at 4°C. Beads were collected and sequentially washed 5 times with 1 ml each of the following buffers: low salt wash buffer (0.1% SDS,1% Triton X-100, 2 mM EDTA, 20 mM Tris-HCl pH 8.1, 150 mM NaCl), high salt wash buffer (0.1% SDS,1% Triton X-100, 2 mM EDTA, 20 mM Tris-HCl pH-8.1, 500 mM NaCl), LiCl wash buffer (0.25 mM LiCl, 1% IGEPAL-CA630, 1% deoxycholic acid, 1 mM EDTA, 10 mM Tris-pH 8.1) and TE buffer. The immunocomplexes were eluted two times by adding a 250 μl aliquot of a freshly prepared solution of 1% SDS, 0.1 M NaHCO_3 _and the cross-linking reactions were reversed by incubation at 65°C for 4 h. Further, the samples were digested with proteinase K (10 mg/ml) at 42°C for 1 h, DNA was recovered by phenol/chloroform extractions, ethanol precipitated using 1 μl of 20 mg/ml glycogen as the carrier, and resuspended in sterile water. For PCR analysis, aliquots of chromatin before immunoprecipitation were saved (Input). PCR was performed to analyze the presence of DNA precipitated by specific antibodies by using the primers reported in Table 1.

Each PCR reaction was performed with 10 μl of the bound DNA fraction or 2 μl of the input. The PCR was performed as follows: preincubation at 95°C for 5', 30 cycles of 1' denaturation at 95°C, 1' annealing at 62°C and 1 min at 72°C, with one final incubation at 72°C for 5'. No-antibody control was included in each experiment.

### Statistical analysis

Data are presented as the mean ± SEM from at least three independent experiments. Statistical analysis was performed by one-way analysis of variance and the Student's t-test. A P value < 0.05 was considered statistically significant.

## Abbreviations

Lef-1: Lymphoid Enhancer binding Factor-1; hOBs: human osteoblasts; hMSCs: human mesenchymal stem cells; FBS: fetal bovine serum; ChIP: Chromatin Immunoprecipitation; HMG: high mobility group; RUNX2: Runt-related transcription factor 2; ALP: alkaline phosphatase.

## Authors' contributions

EL participated in the study design, cloned SLUG promoter, carried out the characterization of osteoblasts, ChIP assays, and experiments with SB216763. TF was responsible for the luciferase assays and helped the ChIP assays. ET performed the western blot assays, RT-PCR analysis and contributed to cell culture experiments. LP was responsible for the isolation and characterization of mesenchymal stem cells from Wharton's Jelly. AP and SP collected bone samples during surgery interventions. RG contributed to data interpretation and provided useful suggestions. RP designed the studies, analyzed data and wrote the manuscript. All authors helped to draft the manuscript, and to read and approve the final version.

## Supplementary Material

Additional file 1 **Detection of SLUG expression by quantitative RT-PCR in osteoblastic-like cell lines and hOB samples. **The level of SLUG was examined by quantitative RT-PCR in U2OS, SaOS-2, Hobit, CAL72 osteoblastic-like cell lines and in eight hOB samples. MCF7 breast cancer cell line was used as negative control. The cDNA obtained from total RNA was subjected to quantitative TaqMan RT-PCR for SLUG transcript analysis. The experiments were carried out in triplicate, the expression levels were normalized on the basis of GAPDH expression and results of the experiments are reported as relative mRNA expression levels. ΔΔCt method was used to value the gene expression; standard error of the mean (SEM) was calculated. Click here for file

Additional file 2 **Treatment of osteoblastic-like cell lines with the glycogen synthase kinase (GSK-3β) inhibitor, SB216763. **The levels of SLUG expression was examined by quantitative TaqMan RT-PCR in U2OS, SaOS-2, Hobit, CAL72 osteoblastic-like cell lines treated with SB216763 (10, 25 and 50 μM) or with the only vehicle (-), up to 3 days. Click here for file
