# Supplementary material for: SLUG: a new target of lymphoid enhancer factor-1 in human osteoblasts
Source: BMC Mol Biol. 2010 Feb 3;11:13. doi: 10.1186/1471-2199-11-13 (PMC2834684; doi:10.1186/1471-2199-11-13)
Supplement: Additional file 1 — Detection of SLUG expression by quantitative RT-PCR in osteoblastic-like cell lines and hOB samples. The level of SLUG was examined by quantitative RT-PCR in U2OS, SaOS-2, Hobit, CAL72 osteoblastic-like cell lines and in eight hOB samples. MCF7 breast cancer cell line was used as negative control. The cDNA obtained from total RNA was subjected to quantitative TaqMan RT-PCR for SLUG transcript analysis. The experiments were carried out in triplicate, the expression levels were normalized on the basis of GAPDH expression and results of the experiments are reported as relative mRNA expression levels. ΔΔCt method was used to value the gene expression; standard error of the mean (SEM) was calculated. [file 1471-2199-11-13-S1.PPT]

## Slide 1
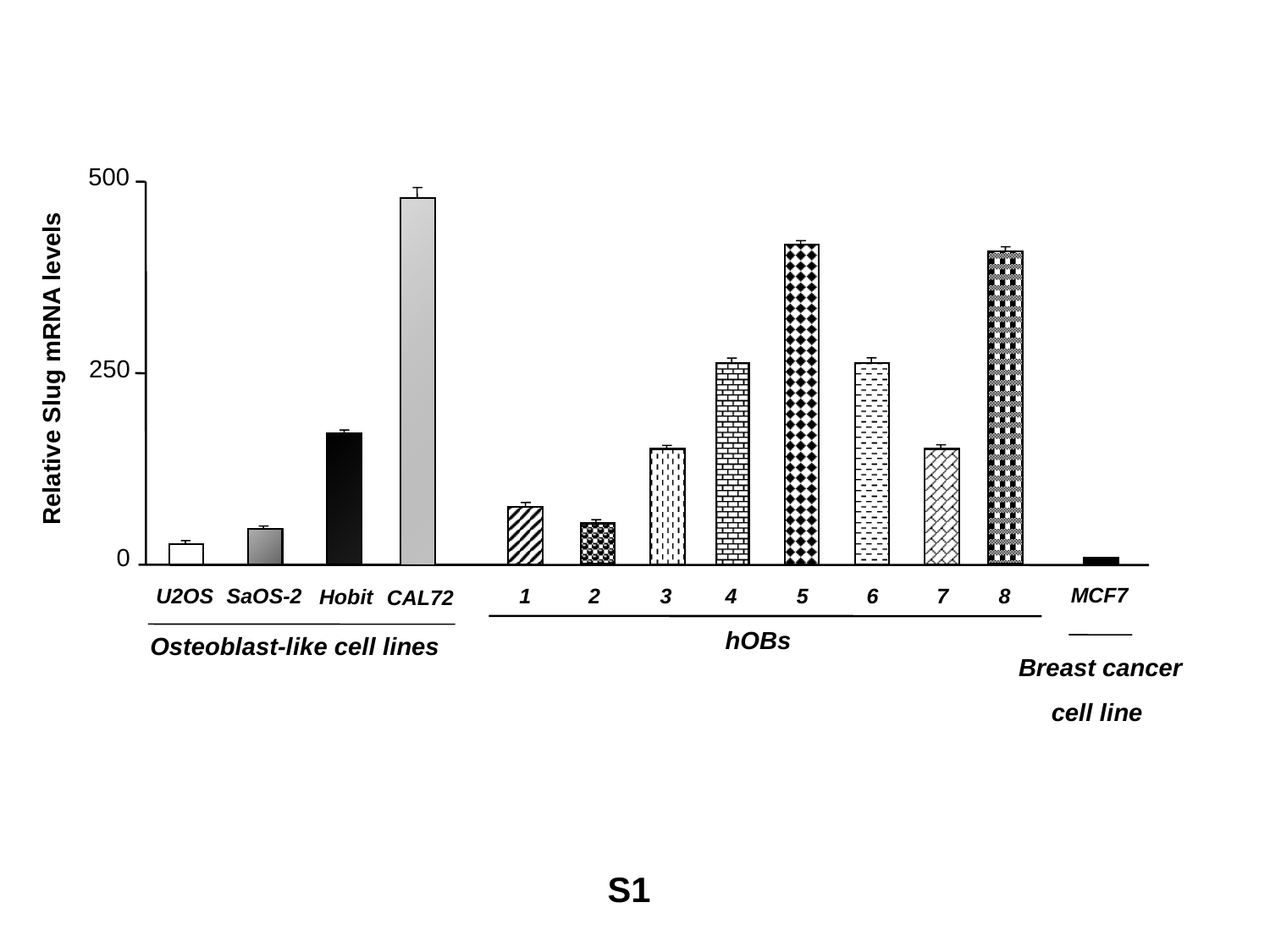

500
Relative Slug mRNA levels
250
0
1
2
3
4
5
6
7
8
MCF7
U2OS
SaOS-2
Hobit
CAL72
hOBs
Osteoblast-like cell lines
Breast cancer
cell line
S1
