# Supplementary material for: SLUG: a new target of lymphoid enhancer factor-1 in human osteoblasts
Source: BMC Mol Biol. 2010 Feb 3;11:13. doi: 10.1186/1471-2199-11-13 (PMC2834684; doi:10.1186/1471-2199-11-13)
Supplement: Additional file 2 — Treatment of osteoblastic-like cell lines with the glycogen synthase kinase (GSK-3β) inhibitor, SB216763. The levels of SLUG expression was examined by quantitative TaqMan RT-PCR in U2OS, SaOS-2, Hobit, CAL72 osteoblastic-like cell lines treated with SB216763 (10, 25 and 50 μM) or with the only vehicle (-), up to 3 days. [file 1471-2199-11-13-S2.PPT]

## Slide 1
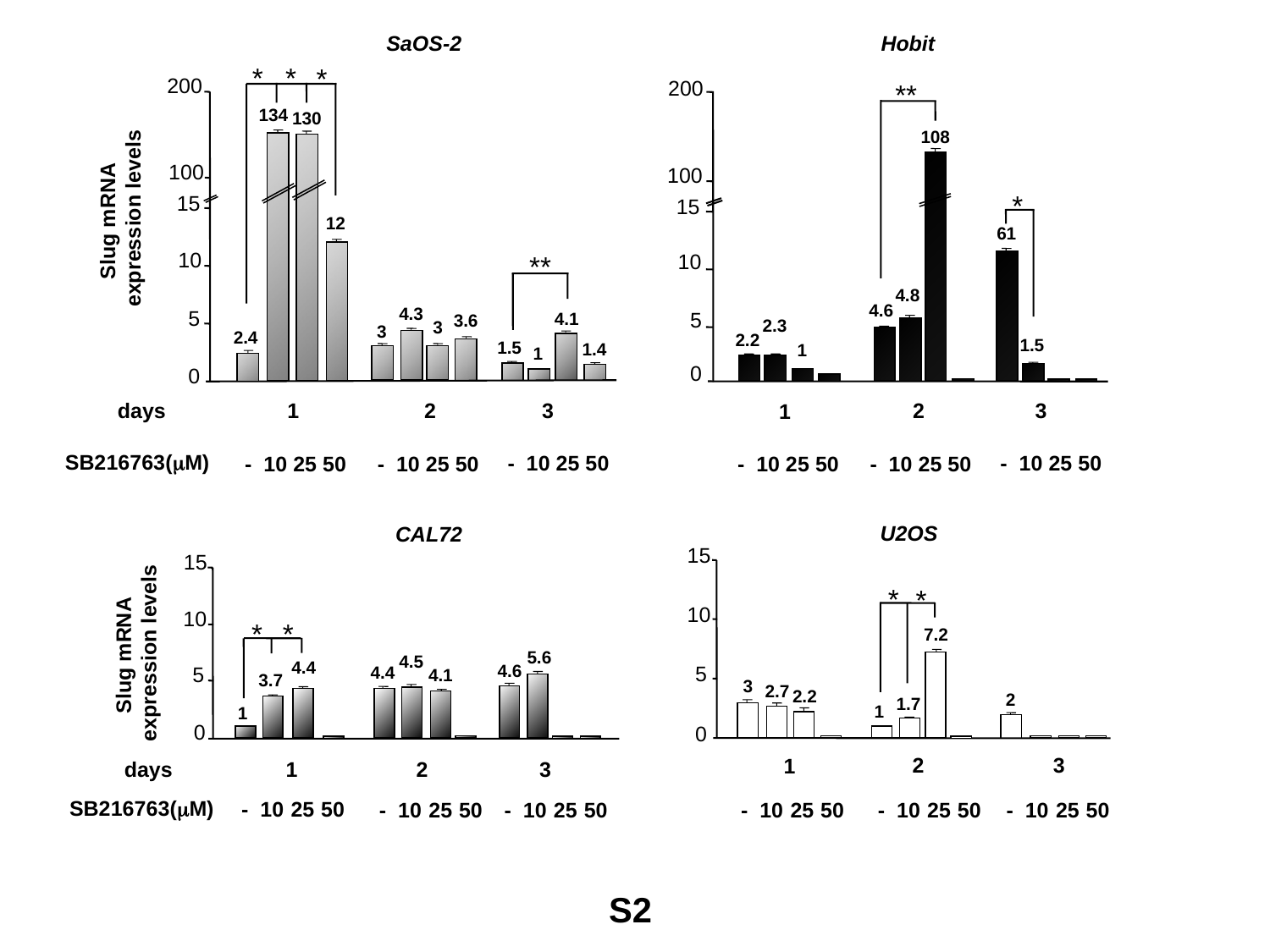

SaOS-2
Hobit
*
*
*
**
200
200
134
130
108
100
100
*
Slug mRNA
 expression levels
15
15
12
61
**
10
10
4.8
4.6
4.3
4.1
3.6
5
5
2.3
3
3
2.4
2.2
1.5
1.5
1.4
1
1
0
0
2
3
days
1
2
3
1
SB216763(μM)
 - 10 25 50
 - 10 25 50
 - 10 25 50
 - 10 25 50
 - 10 25 50
 - 10 25 50
U2OS
CAL72
15
15
*
*
10
10
*
*
7.2
Slug mRNA
expression levels
5.6
4.5
4.4
4.6
4.4
4.1
5
5
3.7
3
2.7
2.2
2
1.7
1
1
0
0
2
3
1
2
3
1
days
SB216763(μM)
 - 10
25
50
 - 10
25
50
 - 10
25
50
 - 10
25
50
 - 10
25
50
 - 10
25
50
S2
